# Risk-appropriate regulations for gene-editing technologies

**DOI:** 10.1080/21645698.2023.2293510

**Published:** 2024-01-12

**Authors:** Graham Brookes, Stuart J Smyth

**Affiliations:** a PG Economics Ltd, Dorchester, UK; b College of Agriculture and Bioresources, University of Saskatchewan, Saskatoon, Saskatchewan, Canada

**Keywords:** Gene editing, Regulation, GMOs, Global Biodiversity Framework, risk appropriate, evidence based, Sustainable Development Goals

## Abstract

This paper explores the scope for the newly emerging technologies, based on gene editing (GE) contributing to addressing the global challenges that we face. These challenges relate to food security, climate change and biodiversity depletion. In particular, it examines the science and evidence behind the most appropriate forms of agricultural production to meet these challenges, the targets set in the Global Biodiversity Framework (GBF) agreed to at the end of 2022 and the possible role of GE technologies in contributing to meeting these targets. It then examines the most risk-appropriate regulatory environment required to best facilitate the adoption of GE technology, drawing on the experiences of the impact of regulatory systems for other innovations used in agricultural and food production systems such as genetically modified organisms (GMOs).

**Note on Definitions**. *This paper discusses regulatory efficiency and the most risk-appropriate regulation of technologies generally referred to as gene editing. It does not explore or discuss the detailed types or categories of gene-editing techniques and how these might or might not be classified as gene editing or as genetic modification for regulatory purposes. For more detailed scientific descriptions of gene-editing types and techniques, as well as comparable descriptions for genetic modification, see, for example, Royal Society (undated) Jargon buster updated v3 (royalsociety.org)*^1^.

## Global Challenges Facing Agriculture and Biodiversity

Providing the world’s population with access to adequate quantities of food, at reasonable prices, whilst not contributing to destroying the Earth’s climate and many species of plant and animal life is one of the biggest challenges facing us. The global target to better protect nature by addressing declining levels of global biodiversity was formalized at the end of 2022 in the development of the Global Biodiversity Framework (GBF). At the 15^th^ Conference of the Parties to the Convention on Biological Diversity (COP-15), held from December 7–19, 2022 in Montreal, Canada, and following nearly 2 weeks of discussions and negotiations on a wide range of issues and topics relating to biodiversity, the Kunming-Montreal GBF^a^^a^The full document is available at: https://www.cbd.int/doc/decisions/cop-15/cop-15-dec-04-en.pdf. was agreed to by 188 Parties at the meeting. The agreement set a global target to conserve and manage 30% of the planet’s land and water by 2030, known as the ’30 by 30’ deal. The GBF set out four main goals, all of which have 2030 as their timeline: first, to expand and where possible restore, natural ecosystems; second, ensuring that nature’s contributions to humanity are not declining; third, sharing the monetary and non-monetary benefits of genetic resources, especially through the use of digital sequence information; and fourth, ensuring that all Parties have the means to successfully implement the GBF.^2^ The GBF establishes 23 targets to reduce threats to biodiversity by 2030.

There is no uniform agreement on how best to deliver on these four main goals. Many environmental non-governmental organizations (NGOs) in attendance at the 15^th^ Conference of the Parties (COP-15) openly advocated at the conference for global returns to zero synthetic chemical or fertilizer input use, widespread adoption of organic and less extensive agricultural production systems and the rejection of modern innovative agricultural production practices and processes as the keys to promoting increased biodiversity and protecting nature (e.g. Agroecology Now, Desertif’cations).^3,4^ They further advocated the allocation of more resources to support an accelerated transition away from ‘intensive’ forms of agriculture in favor of more extensive production systems and the complete removal of some land out of agriculture in a largely ‘land-sharing’ approach to agricultural support.

The major problem with this NGO-driven vision is that science and evidence do not support it. As Balmford^5^ points out, we start from a baseline that ‘*the vast majority of species are strongly dependent on natural habitats, and natural vegetation is of disproportionate value in sequestering and storing carbon’* – in other words, agriculture *per se* is not good for nature. This means that the world has to find production systems and land uses that meet our food needs at least cost to nature. As Balmford^5^ further concludes, *“a land sharing and sparing approach provides such a quantitative approach for delivering this.”* In this compartment-based approach to land use, some land (the largest share) currently in agricultural production should aim to maximize yields, allowing some land not required for food production to be used to retain or restore areas of natural habitat outside agriculture, with a relatively small third compartment reserved for lower yielding/less intensity farming because some farmland species fare better under lower-intensity/extensive farming systems.^6,7^

The problem with focusing on the land-sharing approach is that it seeks to deliver food production and conservation at the same time by reducing input use and production intensity and creating small-scale habitats such as unsprayed field margins and small patches of woodland. Typically, a land-sharing approach results in lower yields, so that at a national or global level, a greater land area is required to produce the same amount of food. Balmford and Bateman^8^ recently warned that countries such as the UK and the EU, which are adopting mostly land-sharing policies with the intention of reducing farming’s environmental impact – including agri-environment schemes, rewilding and organic farming – may unwittingly accelerate global biodiversity loss and negative climate impact by reducing domestic production and driving up food imports unless corresponding policy action is taken to deliver yield increases elsewhere to maintain and expand domestic food production. In a follow-up article,^9^ the same authors wrote *“Figuring out how to feed, clothe and power 10 billion people without causing mass species extinction and wrecking the climate is this century’s greatest challenge. The scientific evidence in support of land sparing is compelling. So why is it not the dominant policy approach today?”*

There is also a rapidly increasing volume of peer-reviewed evidence that soundly refutes arguments against the use of intensive agricultural production systems utilizing the latest innovations.^10–13^ The importance of innovation in increasing food production was also highlighted in the Organization for Economic Cooperation and Development’s^14^ report, which showed that since 1960, global food production has increased by 390%, while the amount of land used to produce food has only increased by 10%. Agricultural innovations over the past 50 years have therefore delivered substantial benefits, especially those resulting from the selectively bred, high-yielding varieties of rice, wheat and maize in the 1960s ^15^ and, since the mid-1990s, the adoption of genetically modified (GM) crops. The benefits of widespread GM crop use include increased yields, ^11^ reduced environmental impacts associated with pesticide use,^16^ reduced greenhouse gas (GHG) emissions^17^ and lower biodiversity impacts.^18^ If GM crops had not been commercialized in the mid-1990s, it is estimated that 23.4 million hectares of additional cropland would have been required to produce the global volumes of canola, cotton, maize and soybeans produced in 2020.^19^ This is an area equivalent to the combined agricultural area of the Philippines and Vietnam.

The science and evidence therefore tell us that the best way forward for future land use and agricultural production systems is to recognize that a combination of high yield farming, natural habitat and lower-yielding farming systems is required and that where agriculture takes place, there is a need to sensibly combine production methods and techniques used in both high- and lower-intensity production systems and embrace (not reject) the adoption of new innovations and technology like improved plant genetics, digital agriculture and precision farming.^b^^b.^Digital agriculture refers to the use of digital technology to accelerate and scale up the use of new or improved production techniques after storing, analyzing and sharing data. Precision farming refers to the use of satellite positioning data and remote sensing to optimize farm management decision taking and the use of inputs.

Returning to GBF, simply put, global biodiversity will not improve without investments into innovative products and technologies that are capable of achieving the 23 proposed targets. Regulatory burdens and regulations that are risk inappropriate have contributed to limiting higher crop and food production over the past 30 years that might otherwise have been achieved by discouraging the commercial adoption of productivity-enhancing technologies in many parts of the world. For example, in 2020, only 1.8% of the total global GM crop planted area was in Africa and the GM crop area found in the European Union (EU) was even smaller at 0.1% of the global GM crop area.^19^ In the EU, the GM crop is limited to a single insect resistant trait found in some maize varieties grown only in Spain and Portugal, which was first approved for cultivation in 2003. This GM maize trait is no longer present in maize varieties grown in the main GM maize adopting countries in 2020 having been replaced many years ago by superior performing GM traits.

## GE Technology and Its Potential Contribution to Achieving the Goals of the GBF

The use of new innovative technologies stemming from gene editing (GE) has the potential to make an important contribution to delivering more productive agriculture and contributing to reducing the environmental and biodiversity-impact footprints of the land devoted to agriculture, as well as facilitating some land currently in agriculture reverting to semi and natural habitats. Therefore, in the context of GBF, which ones of the 23 established targets have implications for the possible utilization of GE technologies or relate to the possible application of genomic innovation? The following discussion connects the existing literature for opportunities to make significant contributions to achieving specific targets.

### Target 6 – Controlling Invasive Alien Species

The objective of Target 6 is to *‘eliminate, minimize, reduce and or mitigate the impacts’* upon biodiversity resulting from invasive species by at least 50% by 2030. Invasive species are species that are not indigenous or native to a specific area or region, whose presence can cause economic and/or environmental damage.^20^ The difference between invasive species and non-native species that are intentionally adopted, is that the non-native adopted species do not result in economic and/or environmental harms or damage. Examples would be potato and corn adoption and production in Europe, as both species originated in the Americas. Similarly, wheat adoption in the Americas would represent the adoption of a non-native, non-harmful species.

Invasive species are capable of causing substantial environmental damage, biodiversity loss and even species extinction. The uncontrolled spread of an invasive species can damage local environments and ultimately reduce the local biodiversity as some native species lack the ability to compete. It is estimated that in 2019, the economic cost of invasive species was $423 billion.^21^

Control of invasive species has been extremely difficult and eradication virtually impossible. In terms of controls for invasive plant species, chemicals are the common control mechanism, while in some instances, removal of plants by hand has also been undertaken. Efforts to control invasive animals include poisoning and shooting. Advances in genetics potentially offer new solutions, using gene-editing technology to create sterile males and release them into the wild.

For plants, hybrid technology has been used to create sub-fertile varieties since the first use in corn in the 1930s. Hybrid varieties can be replanted and will germinate, however, yields will be dramatically lower than occurred in the parent. Invasive weeds often exist outside of crop producing fields, such as in a nature preserve or protected habitat area, allowing them to spread due to the lack of control. Invasive weed species could be developed that are sterile and released in heavily infested areas as a means of control that does not involve chemical applications, as it is often the case that invasive plant species exist in areas that are difficult to spray, such as in sensitive eco-regions or forested areas. The spread of sterile pollen would contribute to reduced weed populations within a few seasons.

Sterility could be induced in invasive animals, as a means of population control. In normal inheritance, an allele for each gene from each parent is passed to the offspring, however, using gene drives, it is possible to edit the allele, thereby increasing the spread of the edited allele in the population.^22^ Targeting and replacing a female fertility gene with a gene drive can be used to prevent females from reproducing. Applying gene-editing technologies will not provide an instantaneous solution but may be part of a long-term strategy.

Given that previous control methods have experienced, at best, marginal success, the application of sterility through gene-edited gene drives offers an innovative solution to protecting biodiversity from invasive species. While application of this innovative approach may not result in 50% reductions of invasive species by 2030, given the time required to implement the solution in animals, certainly 2040 offers a realistic timeline for measurable population reductions. The release of gene-edited invasive weed species would additionally require risk-appropriate regulation, meaning that no additional risk assessment would be required, provided no foreign DNA is present in the final variety and proof-of-concept was robustly established. To reduce the global costs of invasive species, new innovative strategies are required, such as those discussed, as evidence confirms that previous and current strategies have had, at best, marginal success in terms of reducing both the populations of invasive species, as well as restricting the ability of their population to spread into new territory. For gene-edited plant and animal varieties to make significant contributions to mitigating the impacts of invasive species, risk-appropriate regulation is of fundamental importance.

### Target 7 – Nutrient Loss and Chemical Use

The objective of Target 7 is to reduce the potential harms to biodiversity from nutrient loss by at least 50% and to reduce chemical use and the impacts of chemical use by at least 50%, without negatively impacting food security by 2030. This target clearly states that science has to serve as the basis for assessing changes in both the rates of fertilizer and chemical use. This target is identical to the European Union’s Farm to Fork Strategy that calls for a similar 50% reduction in both the application of agricultural chemicals and the environmental impacts of these chemicals.^23^

The adoption of GM crops has made important advancements in the reduction of the environmental impact associated with pesticide active ingredient applications to crop production. Smyth et al.^16^ assessed the impacts of reduced chemical use, finding that the environmental impact of herbicides applied to canola in western Canada dropped by 53%, following the first decade of GM canola production from 1997 to 2006. Extending this study, Lika et al. (forthcoming a)^24^ found further reductions in the environmental impacts from in-crop herbicide application in Saskatchewan. This research quantified the reduced environmental impact at 65% from 1991–1994 to 2016–2019 and that the amount of herbicide active ingredient applied per acre dropped by 45%.

Klümper and Qaim^11^ assessed the data from 147 studies on the farm-level impacts from GM crop adoption, finding a 37% reduction in the use of chemical pesticides following adoption. Brookes^25^ estimated that between 1996 and 2020, GM crop use reduced the application of crop protection products by 748.6 million kilograms of active ingredients, a global reduction of 7.2% in the area planted for GM crops. As a result, farmers who grow GM crops reduced the environmental impact associated with their crop protection practices by 17.3%, as measured by the indicator the Environmental Impact Quotient. The application of GE technologies to crop varieties other than canola, corn, cotton and soybeans (the four major GM crops) has the potential to lead to further reductions in both pesticide applications and the associated environmental impacts of the chemicals that are applied.

While it is important to reduce nutrient loss in watersheds, this is being partly accomplished through the replacement of tillage-based production systems in favor of reduced and no tillage systems. In turn, this reduces soil erosion, so that nutrient loss is reduced. The adoption of herbicide tolerant GM crop technology by delivering more efficient weed control systems has facilitated this move away from plow-based systems in North and South America and helped farmers remain in no and reduced tillage systems. If a 50% reduction was directly applied to the application of fertilizers used to provide sufficient nutrients to produce crops, this would likely have devastatingly negative impacts on crop production. An estimate of a proposed 30% nitrogen fertilizer emission reduction in Saskatchewan has been estimated to cost farmers C$48 billion in farm revenue between 2023 and 2030.^26^ Lika et al. (forthcoming b)^27^ found that in Saskatchewan, whilst total fertilizer use increased by 102% between 1991–1994 and 2016–2019, fertilizer use efficiency, that is the volume of crop produced per acre, per pound of fertilizer applied only increased by 29%. The impact of introducing bans on the use of fertilizers in agriculture has also been shown from the experience of Sri Lanka where the government decided to ban the import and use of synthetic fertilizers and pesticides as part of a planned transition of the country’s agricultural sector to organic agriculture in 2021. As a result of the ban, the yields of the main crops (rice, tea, rubber, coconut and spices) fell by between 30% and 50% with economic losses estimated at US$ 425 million.^28^ This highlights the importance of fertilizer use to maintaining and enhancing the global production levels required to meet global food security but in as efficient a way as possible. More efficient use of fertilizers is key, not blanket reductions in fertilizer use targets. Gene-editing research is one technology being used to target increased nitrogen use efficiency that could reduce/replace fertilizer use.^29,30^ Nitrogen-fixing soil microbes can be edited to increase nitrogen fixation in crops, allowing for greater in-crop yield consistency, as well as increased overall yields.^31^

### Target 10 – Agriculture and Sustainable Biodiversity

The objective of Target 10 is to ensure that agriculture, aquaculture, fisheries and forestry are sustainably managed, particularly through the application of biodiversity-friendly practices that result in increased productivity and food security. Agriculture and aquaculture have experienced research and development investments and the commercialization of products that have greatly focused on improved sustainability management. Fisheries and forestry have currently had efforts targeted at increasing sustainable management, with mixed results. The management of wild fish stocks has been successful with some species, yet has been more challenging with others. Forestry sustainability has had a mixture of approaches from logging bans in sensitive areas, to increased replanting in forested areas.

Over the past 30 years, agriculture has made tremendous sustainability advancements, particularly from the adoption of GM crops, that have driven the transition of land management practices from being tillage intensive to having removed nearly all tillage practices.^32^ The application of gene-editing technologies demonstrates the ability to further contribute to improved sustainability of crop production through improved nutrient use efficiency^33^ and water use efficiency.^34^ Improving nutrient use efficiency results in a decreased nutrient need; therefore, there is a reduced potential for nutrients to move from cropland into local watersheds. Improved water use efficiency enables crops to better withstand periods of reduced precipitation without experiencing significant yield losses. Increases in the use efficiency of both factors will make significant contributions to reducing the impact that agriculture production has on biodiversity, as well as contributing to yield enhancements.

Complimenting yield increase research is the research concentrated on improving the nutritional content of food crops through the application of gene-editing technology. Substantial research has been undertaken on improving nutritional quality that includes enhanced protein (canola, corn, potato, rice and wheat); increased oils and fatty acids (canola, corn, rice and soy); improved carbohydrates (corn, potato, sugar beet and soy); increased vitamins (potato, rice, strawberry and tomato) and increased mineral availability (lettuce, rice, soy, corn and wheat).^35^ Numerous micronutrients, such as iron, zinc, selenium, magnesium, calcium and iodine, and vitamins like provitamin A and folate play essential roles in the healthy development of children and the nutrition of nursing women. As well, vegetables are increasingly the focus of research addressed at increasing these vital nutritional compounds.

Gene editing has also been applied to aquaculture development, with the commercialization of two varieties of gene-edited fish in Japan.^36^ Red sea bream and tiger puffer fish have both been gene edited to increase the growth of more muscle tissue, 20% and 90%, respectively. This results in less feed required for the fish to reach market weight, where they are sold fresh online for sushi consumption. Laboratories in China, Norway and the USA are all investing into using gene editing in the aquaculture industry to improve production sustainability.^37^ This research has focused on a variety of factors, including increased disease resistance, improved reproduction, enhanced growth rates and increased omega-3 metabolism.

Gene editing also has the potential to play an important role in the improvement of forest health through the development of insect resistant trees. In many parts of the world, forest fires have increased in recent years. Part of the reason for this is that one of the most significant results from climate change in the forestry sector has been the migration of insects, like the spruce wood budworm and mountain pine beetle. Warmer winters have allowed both of these insects to spread further north into areas where they previously could not exist, resulting in tens of millions of acres of dead forest. Natural Resources Canada estimates that half of the commercial forest in British Columbia have been killed by mountain pine beetles over the past 25 years.^38^ With such vast tracts of dead forest, forest fires grow in size more rapidly than previously resulting in out of control fires, whereas with healthy living forests, the fire could be more easily contained.

## Delivering the Innovation to Help Meet the Global Challenges: The Importance of Risk-Appropriate Regulation

Creating a suitable and enabling environment for new innovation adoption and use in global agriculture requires a regulatory framework that facilitates and encourages innovators to bring new technologies to market. In contrast, an inappropriate regulatory framework discourages innovators from bringing new products to market and reduces the scope for adoption.

In the rest of the paper, we explore the appropriate regulations and assessments made to determine how enabling or innovation friendly different regulatory systems used in agricultural and food product production systems has been. The findings of this review are then used to discuss what a risk-appropriate regulatory environment might be for the application of products derived from emerging technologies like GE.

### What Is Appropriate Regulation?

In trying to assess the extent to which regulation is ‘innovation-friendly’ or not, it is first instructive to look at the purpose and categories of regulation and which category of regulation is of greatest relevance to GE technology. Regulation is a set of rules that oversee the economic activities of anyone operating in a market (businesses, individuals) in order to achieve social goals such as those related to health, safety, labor use, as well as the minimization of environmental damage and pollution. Blind ^39^ usefully classified regulation into a number of categories:

- Economic regulation which tries to avoid market failure;
- Competition regulation: which provides a framework for competition, aiming to maximize competition in markets and minimize barriers to entry to a market;
- Social regulation, which targets the removal of externalities such as pollution and environmental damage;
- Labor and consumer safety regulation which aims to protect the health and safety of consumers and workers;
- Institutional regulation: which covers product liability (which overlaps with health and safety regulation) and the definition of intellectual property rights which are important for encouraging innovation.

Each of these categories of regulation target avoidance or correction of some form of market failure, and their success or failure depends on the ability to correct the market failures relative to the costs that the regulation imposes on operators in the market. In the context of regulation of new innovation technologies that may stem from GE, the primary categories of regulation this falls into are social regulation and consumer safety regulation. These regulations aim to protect consumers and animals from possible health risks that could arise from consumption of products that contain or are derived from GE technology and to protect society and the environment from possible adverse impacts or ‘pollution’ that might arise from their use in agricultural production systems.

The ideal ‘innovation-friendly’ form of GE regulation will limit the impacts of compliance costs and promote/encourage or provide incentives to innovate mainly by reducing risk and uncertainty for the developers of products containing or derived from GE technology. The actual impact of regulation on innovations using this technology will depend on the extent to which GE innovators have to divert resources away from the productive activities of developing profitable new products (e.g., new seeds with desirable traits that improve the productivity and quality of crops) to meet the regulatory compliance requirements. However, this does not mean that innovators view regulation purely in a negative light because there are costs associated with compliance. From the perspective of new product developers using GE, regulation of the technology is important because it plays a positive role in ensuring consumer confidence in products with costs associated with meeting regulatory requirements considered as a necessary and important component of bringing a new innovation to market. In addition, regulation can have an incentive effect by providing innovators with greater market clarity and risk/uncertainty reduction. For this to happen, innovators expect regulation to be science-based, have a transparent process, be predictable, and show clearly how risk is being assessed. If regulatory systems deliver on all of these expectations, this minimizes uncertainty and risks associated with regulation, with the costs of regulation being ‘calculable’.

## Assessing whether a Regulatory System Is Appropriate and ‘Innovation Friendly’

Delivering this optimal regulatory system is the challenge, especially when developing a system to regulate a new technology like GE. However, much can be learned from the experiences of regulatory system operation and its impact on innovations derived from similar technologies like genetic modification (GM) over the last 25 years, as well as innovations and regulations of new food products such as those classified as novel foods or foods that have health claims.

One way of assessing the impact of regulation on innovation is to explore the costs of meeting regulatory requirements on the expected returns that a business might reasonably expect to earn if it brings a new product to market. A decision on whether or not to innovate is inherently risky given a number of unknown factors, including the outcome of prospective research, market uptake for any new product and the behavior of competitors. One particularly important consideration for any company assessing the viability of investment is the anticipated return on this investment, which is determined to a significant extent by the time taken for a new product to come to market. Regulatory procedures, both how they are designed and how they are implemented, are therefore of utmost importance to a company’s innovation strategy.

Quantifying the costs associated with bringing a GM crop trait to commercialization inclusive of the costs of meeting regulatory requirements has been the focus of many researchers over the last 25 years. Phillips and Williams^40^ have painstakingly summarized much of this research in their meta-analyses of GM crops regulatory approval costs. A lot of the studies cited were conducted in the first decade of this century (2000–2010) and suggested an average cost of regulatory compliance for a single new crop trait seeking regulatory approval in a single market to be $7.8 million (range $0.53 million to $14.8 million). As most GM crop trait innovations were targeted at approval in more than one country for planting and several (export) markets for importation and use, the aggregate regulatory cost suggested in this paper was estimated at $55 million (assuming two country approvals for planting and five for importation and use). This compared with industry estimates for the period 2008–2012 of about $35.1 million (Phillips MacDougal for CropLife International (2011)).^41^

Many of these studies also examined the time taken to complete the regulatory approval process and its impact on costs and returns on investment. For example, Bayer, Norton and Falck-Zepeda^42^ estimated that a one-year delay in the regulatory approval process would potentially reduce the net present value by between 12% and 36% for two crop-specific traits that they were assessing at that time (insect-resistant rice and virus-resistant tomato). The net impact of the ‘upfront’ research and development costs for developing crop biotechnology trait innovations, and the long lead time involved before these innovations can be commercialized (inclusive of both the cost and time of regulatory compliance) has therefore not surprisingly resulted in a low number of players successfully bringing GM crop traits to market over the last 25 years. As the Phillips MacDougal 2011 study^41^ illustrates, these have been large multi-national seed and agro-chemical companies. This can be confirmed by an examination of the GM crop plantings in 2020^19^ where 98% of the total GM crops contained traits originally commercialized by the four main multi-national biotech seed companies. They have been the only organizations able to fund the large sums required to complete the regulatory approval process (the 2011 Phillips MacDougal study estimated the overall cost of bringing a crop biotech trait to market at $136 million) and to bear the risks associated with regulatory delays and resultant reductions in returns as measured by net present values. This concentration and focus of crop biotechnology innovations in the hands of a small number of multi-national companies has also probably been exacerbated over the last 10–15 years as subsequent analysis shows. For example, an updated industry assessment of the costs of developing new crop biotechnology traits by AgBioinvestor^43^ (for CropLife International) in 2022 estimated the time and cost to develop a new global GM trait to be $115 million in the period 2017–22 (lower than the original study 10 years earlier), but with a longer mean duration to bring it to the point of commercialization of 16.5 years inclusive of the time taken to gain regulatory approval. The regulatory phase accounted for the largest duration in the overall process (37.6% of the total costs compared to a quarter 10 years earlier and over 50% of the total time). Lassoued et al.^44^ found that if a gene-edited crop was commercialized as equivalent to a GM crop, it would cost US$24.5 million and take 14 years. If the gene-edited variety was regulated as equivalent of conventional varieties, not requiring additional risk assessments, the cost drops to $10.5 million and time drops to 5 years.

The risk to investment associated with the uncertainty of how long the regulatory approval process takes to complete was examined further by Smyth, McDonald and Falck-Zepeda.^45^ They explored the link between investment uncertainty and regulatory approval delays for new plant breeding innovations based on GMOs. This research concluded that regulatory decisions that take longer than 6 years create a level of investment uncertainty that results in businesses suspending further technology investments. This research also concluded that regulatory agencies that provide consistent, timely and science-based regulation do not unduly generate levels of investment uncertainty, in contrast to regulatory systems based on the precautionary principle (e.g., the EU), which create a level of regulatory uncertainty that has discouraged investment in GMO-related innovations.

**Figure 1.**
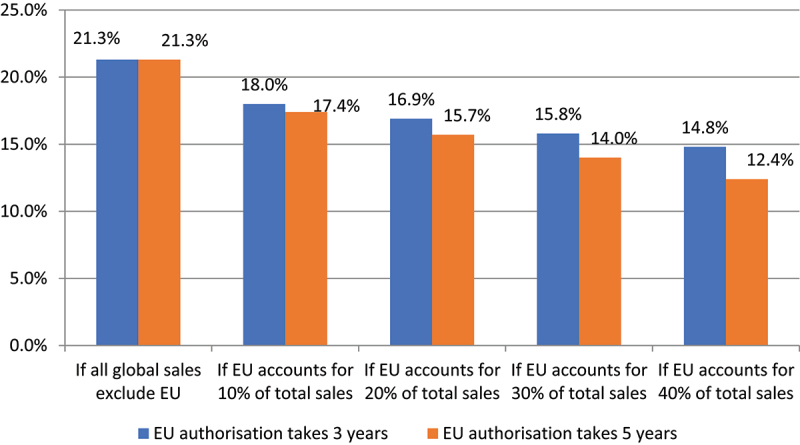
Impact of the EU’s longer authorization process for novel ingredients/products on global returns (% internal rate of return): average returns basis. Source: Brookes and Downes (2017).

The impact of regulatory delays and the uncertainty that this can lead to have also been examined in detail by Brookes and Downes^46^ in relation to the impact of the EU’s regulations of novel food products and health claims on innovation in the specialty food ingredient market. In the case of novel foods, the regulatory procedures for authorizing new products in many countries typically take 12–18 months. A 12–18 month authorization procedure will generally deliver a rate of return of between 16.1% and 25.8% (average 21.3%) within a company’s typical target internal rate of return of 15%–25% and payback time of 4 years. In contrast, the average EU authorization time takes 36 months, which leads to an internal rate of return on investment of between 7.3% and 13.4%. The EU’s regulatory system with its relatively long approval process was considered to be a dis-incentive to bringing new products to this market. In addition, the global nature of the specialty food ingredient market means that a divergence between the EU and other regulatory systems has more far-reaching consequences for companies’ innovation strategies. As EU procedures are typically longer than the authorization processes in other markets, delays incurred through the EU’s regulatory system may significantly inhibit the company’s *global* returns. In such cases, there has been an additional incentive not to bring innovative products to the EU market, to delay European marketing until other markets have been established or even to not bring a product to the entire global market. Here, the ultimate impact on global returns depends upon the importance of the EU market share (Fig. 1) and while the EU market is a key part of expected global sales, the EU regulatory delays may result in a new innovative product not being brought to any market, not just the EU market.

An alternative way in which regulation has impacted on innovation is via its impact on trade, access to and use of agricultural commodities that are derived from new technologies. The use of new seed technologies, notably those utilizing GMOs in the production systems of major exporting countries of these commodities, has been directly influenced by the timing of regulatory approval for importation and use in major importing countries of these commodities. Asynchronous approval of GMOs in different jurisdictions can result in disruption to trade in commodities if approval for importation and use in an importing country is slower than the approval for planting in a major exporting country of that commodity. This is made additionally worse by the near-universal policy of importing countries mandating zero tolerance for the adventitious or low-level presence (LLP) of unapproved GMOs being found in import shipments.

The asynchronous nature of different GMO approval systems allied to the operation of zero-tolerance policies for LLP of unapproved GMOs and their impact on trade have been documented through the ex-post reporting of LLP incidents and impacts on trade. Several were summarized in Kalaitzandonakes et al.,^47^ with the study concluding that LLP incidents cause abrupt, large-scale trade disruptions, induce sustained changes in trade patterns and impose significant economic losses on both importers and exporters. The study also concluded that there is evidence that the asynchronous nature of GMO regulatory approvals and LLP can cause delays to the introduction of some crop seed innovations and hinder the future development of others. Brookes and Kayhan^48^ examined the impact of the asynchronous nature of Turkey’s GMO regulatory approval system for the importation and use of agricultural commodities relative to the timing of cultivation approvals in the main agricultural producing and exporting countries of the world over the period of 2009 to 2020. The study estimated the cost burden incurred by the Turkish agri-food sector as a result of the GMO import regulations came to between $1.53 billion and $2 billion over this period. It also identified that the Turkish GMO regulations had contributed to the domestic agri-food sector having higher costs of production, lower levels of profitability and higher levels of risk and uncertainty associated with accessing raw materials. This resulted in lower levels of investment, value added and employment than would otherwise have occurred if the GMO regulations were based on a more science-based approval system and operated in a transparent and timely manner. Some agri-food processing and manufacturing businesses indicated to the researchers that they have closed down some trading and processing operations in Turkey because of the negative impact on raw material procurement caused by the GMO regulations. In addition, others have chosen to site new investment in other countries because these locations provide for more competitive, stable and less risky access to raw materials.

The potential for trade disruption and impacts on downstream user sectors has also been explored in a number of ex-ante studies. One of the most notable ones was a 2010 analysis of the economic impact of the EU GMO regulations and potential for disruption to trade in and importation of GM-derived commodities and derivatives used in the livestock production sectors undertaken for the Directorate General of Agriculture of the European Commission.^49^ This study concluded that the potential impact of short-term disruption to trade in these products would impose a significant cost burden equal to about €10.5 billion which could be passed down the supply chain to EU consumers of livestock products.

Overall, the findings of the literature exploring the impacts of regulation on investment and innovation are fairly consistent. The contribution of regulatory costs (e.g., the generation of safety or environmental impact data, or clinical/animal trials) to the total costs of research and product development varies by sector. In general, costs associated with meeting regulatory requirements can be up to 50% of the total costs of bringing a product to market. Secondly, the different nature of regulatory systems operating around the world can have a negative impact on innovation through different data requirements, different interpretations of (the same) data and a lack of clarity and transparency on how interpretations and decisions are made. These differences cause delays in the approval process, which negatively impact potential returns on investment not only in a specific market where a regulatory delay occurs but also can negatively impact a new innovation being commercialized in any market. The differences in the nature of regulatory systems also generate uncertainty, which further discourages innovation.

The lack of a global ‘harmonisation’ of regulations across a range of subjects including GMOs, novel foods and health claims is widely perceived as contributing to slowing down the process of bringing products to market in different regions and countries. In particular, the regulatory approval systems in the EU (notably GMOs, but also for novel foods, health claims, food additives and enzymes) are widely perceived to take longer and have a higher degree of regulatory uncertainty than regulatory approval systems in other countries/regions of the world. Much of the ‘blame’ for the negative impact on innovation of the EU’s various regulatory approval systems can be attributed to the EU’s systems being significantly influenced by application of the precautionary principle. This affects the process of risk analysis and management by implying that regulatory approval systems should ensure that risks are avoided even when there is incomplete scientific evidence as to their magnitude or potential effects. This approach allows regulators to make subjective assumptions about the risks associated with new technologies possibly being infinite whilst possible benefits are uncertain.^50^ As a result, evidence standards and limits can lack clarity, the role of science and evidence becomes unclear, and the whole regulatory process lacks transparency. In such a system, evidence of a product’s safety and benefits can be ignored in favor of a ‘best not,’ total avoidance of risk approach. Inevitably, such a system adds cost, delays and uncertainty to the process of new product or technology development and penalizes innovation.

## What Is a risk-appropriate Regulatory System ?

Moving to the subject of regulation of the newly emerging GE technologies, what does constitute a risk-appropriate regulatory system? Given that these technologies are relatively new and there is incomplete evidence of potential impact magnitude or effects, a precautionary approach might be considered as was practiced in some countries and regions of the world in the 1990s when GMOs were first regulated. The science and evidence do not, however, support implementing a ‘best not’ largely risk avoidance regulatory system for GE technology for the following reasons:

- The evidence to date regarding the benefits from biotechnology and its commercialization are consistent, with exceptionally robust supporting data. This evidence will be required where countries are yet to formalize appropriate regulation of gene-editing technologies and already has been drawn on by regulators in Argentina, Australia, Brazil, Canada, Japan and the USA. All of these countries have independently concluded that if no foreign DNA is present in the commercialized product from the application of gene-editing technologies, then no additional risk assessment is required, such as is required for genetically modified crop traits.^51^ This regulatory confirmation will provide all firms, from the large multinational firms to the small and medium-sized enterprises with the greater certainty required to make new agricultural trait and product development investments, knowing that the developed products will be less susceptible to delays from regulations that are not appropriate with the level of risk and founded on a strict version of a precautionary principle;
- The review of the impacts of GMO regulatory systems over the last 25 years shows that regulatory systems where a precautionary principle dominates have created investment uncertainty, increased the cost of bringing products to market, reduced the returns on investment, caused disruption to agricultural commodity trade, added cost to supply chains and discouraged investment;
- The science and evidence show that the best way to address the urgent global food security, climate and environmental challenges that we face is to embrace (not reject) the adoption of new innovations and technology like plant genetics, digital agriculture and precision farming. Returning to the Global Bio-diversity Framework (GBF), simply put, global biodiversity will not improve without investments into innovative products and technologies that are capable to achieving the 23 proposed targets.

In looking at the potential of GE technologies to contribute to addressing the challenges of food security, climate change and biodiversity protection, an important differentiating factor between GE and GM technologies as applicable to crops has an additional potential to affect development and adoption. This relates to how long the research and development process takes. New variety development containing traits developed using GMO technology commonly take at least 12 years before a trait is ready for commercialization and that assumes timely regulatory approval processes. GE technology has the potential to reduce this to 8–9 years and have a significantly lower overall cost of $12–18 million for a single or stacked trait.^52^ These features on their own lower the barriers to entry into the market for products using GE technology and offer the potential for a larger number of (smaller) players to bring GE innovations to the market than has been possible with GM technology. This hypothesis relating to the significant economies in development scale between GE and GM technologies was also highlighted by Bullock, Wilson and Neadeau.^53^

The economies of research and development scale that GE technology offers and the nature of the urgent issues facing the world reinforce the importance of regulatory approval systems for GE technology being appropriate to the risks involved. If the regulation of this technology around the world fails to use science and evidence to appropriately reflect the risks involved and is instead overly precautionary in nature, GE technology will not fulfill much of its promise.

Evidence to date of the impact of regulatory systems in place for assessing and approving GE technology applications in agriculture is currently in an early phase. Argentina was one of the first countries to introduce a specific regulation to approve applications that use GE technology in its agricultural sector back in 2015. The regulation sets out specific scientific criteria to be applied on a case-by-case basis to applications for approval to determine if they should be considered ‘as GMOs’ for the purpose of assessment or considered as conventional technology and not subject to the more onerous, costly and time-consuming process inherent in GM technology approval. Whelan, Gutti and Lema^54^ examined the experiences relating to products derived from GE breeding techniques coming to market in Argentina over the first 4 years of its operation. Their findings largely confirm the conclusions drawn by Bullock et al.^53^ in that GE products have followed a much faster development rate than GM-derived innovations. This rapid rate of adoption was driven by a wider range of developers, led by small and medium-sized enterprises and public research institutions. Finally, the product profiles are more diversified in terms of traits than under GM technology.

## Conclusions

Inefficient and inappropriate regulations result in the creation of barriers to innovation. Regulatory barriers lead to two key outcomes: reduced innovation investments and fewer commercialized products and technologies. For the Global Biodiversity Framework to successfully achieve its targeted objectives by 2030 and for sustainable agricultural production systems to be meaningfully developed and widely adopted, it is of fundamental importance that empirically based regulations, as highlighted in the GBF, as well as underpinning the Sustainable Development Goals (SDGs), be globally enacted. From a practical perspective, this means establishing regulatory systems in which risks are appropriately assessed according to clearly defined scientific criteria and evidence and do not default to an overly precautionary approach in which evidence standards and limits lack clarity, the role of science and evidence becomes unclear, and the whole regulatory process lacks transparency.

Deviations from empirically based regulations and widely accepted scientific standards will delay and stifle the adoption of crucial technologies targeted at contributing to preserving biodiversity and achieving improved agricultural sustainable development. The longer enabling technologies are delayed and stifled, the longer the continuation of negatively impactful practices will last, increasing the overall adverse impacts. Therefore, the solution to meaningful changes lies in shorter peak adoption periods, not longer. For this to happen, all regulations need to be risk-appropriate and evidence-based.
